# Different Effects of Farrerol on an OVA-Induced Allergic Asthma and LPS-induced Acute Lung Injury

**DOI:** 10.1371/journal.pone.0034634

**Published:** 2012-04-26

**Authors:** Xinxin Ci, Xiao Chu, Miaomiao Wei, Xiaofeng Yang, Qinren Cai, Xuming Deng

**Affiliations:** Institute of Zoonoses, College of Animal Science and Veterinary Medicine, Jilin University, Changchun, Jilin, People’s Republic of China; Trinity College Dublin, Ireland

## Abstract

**Background:**

Farrerol, isolated from rhododendron, has been shown to have the anti-bacterial activity, but no details on the anti-inflammatory activity. We further evaluated the effects of this compound in two experimental models of lung diseases.

**Methodology/Principal Findings:**

For the asthma model, female BALB/c mice were challenged with ovalbumin (OVA), and then treated daily with farrerol (20 and 40 mg/kg, ip) as a therapeutic treatment from day 22 to day 26 post immunization. To induce acute lung injury, female BALB/c mice were injected intranasally with LPS and treated with farrerol (20 and 40 mg/kg, i.p.) 1 h prior to LPS stimulation. Inflammation in the two different models was determined using ELISA, histology, real-time PCR and western blot. Farrerol significantly regulated the phenotype challenged by OVA, like cell number, Th1 and Th2 cytokines levels in the BALF, the OVA-specific IgE level in the serum, goblet cell hyperplasia in the airway, airway hyperresponsiveness to inhaled methacholine and mRNA expression of chemokines and their receptors. Furthermore, farrerol markedly attenuated the activation of phosphorylation of Akt and nuclear factor-κB (NF-κB) subunit p65 both in vivo and in vitro. However, farrerol has no effect on the acute lung injury model.

**Conclusion/Significance:**

Our finding demonstrates that the distinct anti-inflammatory effect of farrerol in the treatment of asthma acts by inhibiting the PI3K and NF-κB pathway.

## Introduction

The lung is a very complex immunologic organ and responds in a variety of ways to inhaled antigens, infectious materials or saprophytic agents. Pulmonary disorders can be classified according to the immune responses which they induce. Innate immunity includes the presence of PMNs, an increase in procoagulant activity and the secretion of IL-8, which are important mediators in diseases, such as pneumonia, acute lung injury and its more severe form, acute respiratory distress syndrome (ARDS). On the other hand, adaptive immune conditions like asthma are characterized by adaptive responses which including Th1 or Th2, eosinophils, antibody mediated [1]. Airway inflammation is present in acute lung injuries and asthma caused by different responses [2], [3]. Different results from experiments with infectious and non-infectious mouse airway inflammatory models show a distinct anti-inflammatory role of chemicals that can later be translated for use in the clinic [4], [5].

Mammalian Toll-like receptor (TLR) proteins derive their name from the Drosophila Toll protein, which has ten receptors to date [6]. Some studies have revealed that TLR proteins utilize a similar signaling cascade that ultimately culminates in the activation of NF-κB, activator protein-1, phosphatidylinositol 3-kinase, and mitogen-activated protein (MAP) kinases which play a critical role in pulmonary infectious disease, inflammation and allergic asthma [7], [8]. A better understanding of the roles of the signals regulating lung disease will aid in the promotion of new therapies for the associated symptoms. NF-κB is a key transcriptional factor involved in regulating the expression of proinflammatory mediators, including cytokines, chemokines, and adhesion molecules, thereby playing a critical role in mediating inflammatory responses [9], [10]. PI3Ks is a large family of signaling kinases involved in the inflammatory process. PI3Ks mediate key signal transduction reactions during immune and inflammatory responses and thus represent an attractive target for therapeutic development in various inflammatory diseases [11], [12]. There is increasing evidence that PI3Ks contribute to the pathogenesis of asthma by regulating the expression and activation of inflammatory mediators, inflammatory cell recruitment and immune cell function [13].

**Figure 1:**
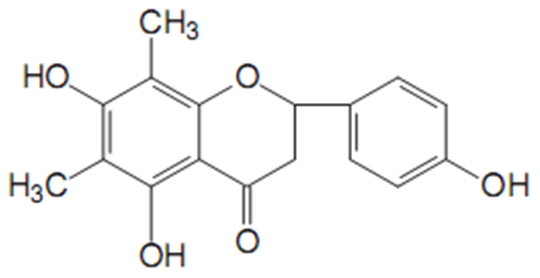
Chemical structure of farrerol.

In folk medicine, plants have long been used to treat a wide range of pathologies, such as cancers, inflammatory diseases and asthma. The flavonoids are a large group of polyphenolic natural products that are widely distributed in higher plants, which are well known to have a variety of therapeutic activities such as against cancers, tumors, inflammation and cardiovascular disease [14]. Some flavonoids isolated from the roots of Rhododendron mucronulatum Turzaninov were reported to be a potential antiinflammatory agents based on the results of dose-dependently inhibited the expressions of inflammatory mediators, NO and PGE2 [15]. Farrerol (structure shown in Fig. 1), a new kind of 2,3-dihydro-flavonoid drug, is also isolated from rhododendron, which belongs to a traditional Chinese herbal medicine [16]. It has been shown to have the anti-bacterial activity, but no details on the anti-inflammatory activity [17].

Inflammation is a hallmark of many human diseases, including pulmonary inflammatory diseases (e.g., chronic obstructive pulmonary disease, and asthma), infectious diseases and cancer. Steroids and cyclooxygenase inhibitors have long been used as the main therapeutical anti-inflammatory agents, but they are frequently associated with significant detrimental effects in patients [18], [19]. Thus, there is an urgent need for the development of unique anti-inflammatory drugs. An understanding of the airway inflammation in the lung in infectious and non-infectious lung diseases is critical to the development of new and innovative therapies for allergic and inflammatory lung disease.

## Results

### Farrerol Suppresses Ovalbumin-induced Inflammatory Cell Recruitment

**Figure 2:**
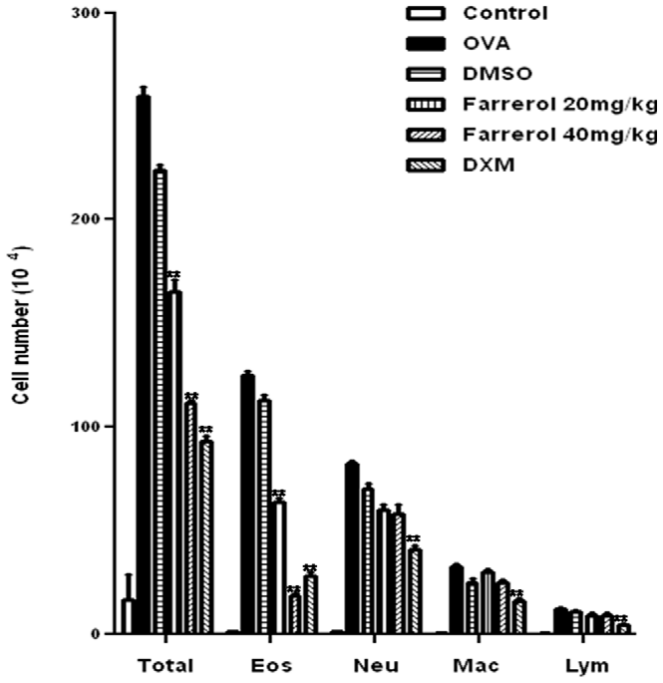
Effects of farrerol on OVA-induced inflammatory cell recruitment and mucus hyper-secretion. Inflammatory cell counts in BALF obtained from sensitized mice 24 h after the last farrerol treatment. Differential cell counts were identified eosinophil (Eos), macrophage (Mac), neutrophil (Neu) and lymphocyte (Lym).

The inflammatory cell levels (i.e., total cells, eosinophils, neutrophils, macrophages and lymphocytes) in the bronchoalveolar lavage fluid were significantly elevated in OVA-challenged mice versus control mice. Farrerol (20 and 40 mg/kg) decreased the total cell number and eosinophils in a dose-dependent manner (**Fig. 2**), as compared with the dexamethasone control, but only slightly decreased the number of infiltrating neutrophils and macrophages.

### Farrerol Suppresses Airway Inflammation, Goblet Cell Hyperplasia and Mucus Production

**Figure 3:**
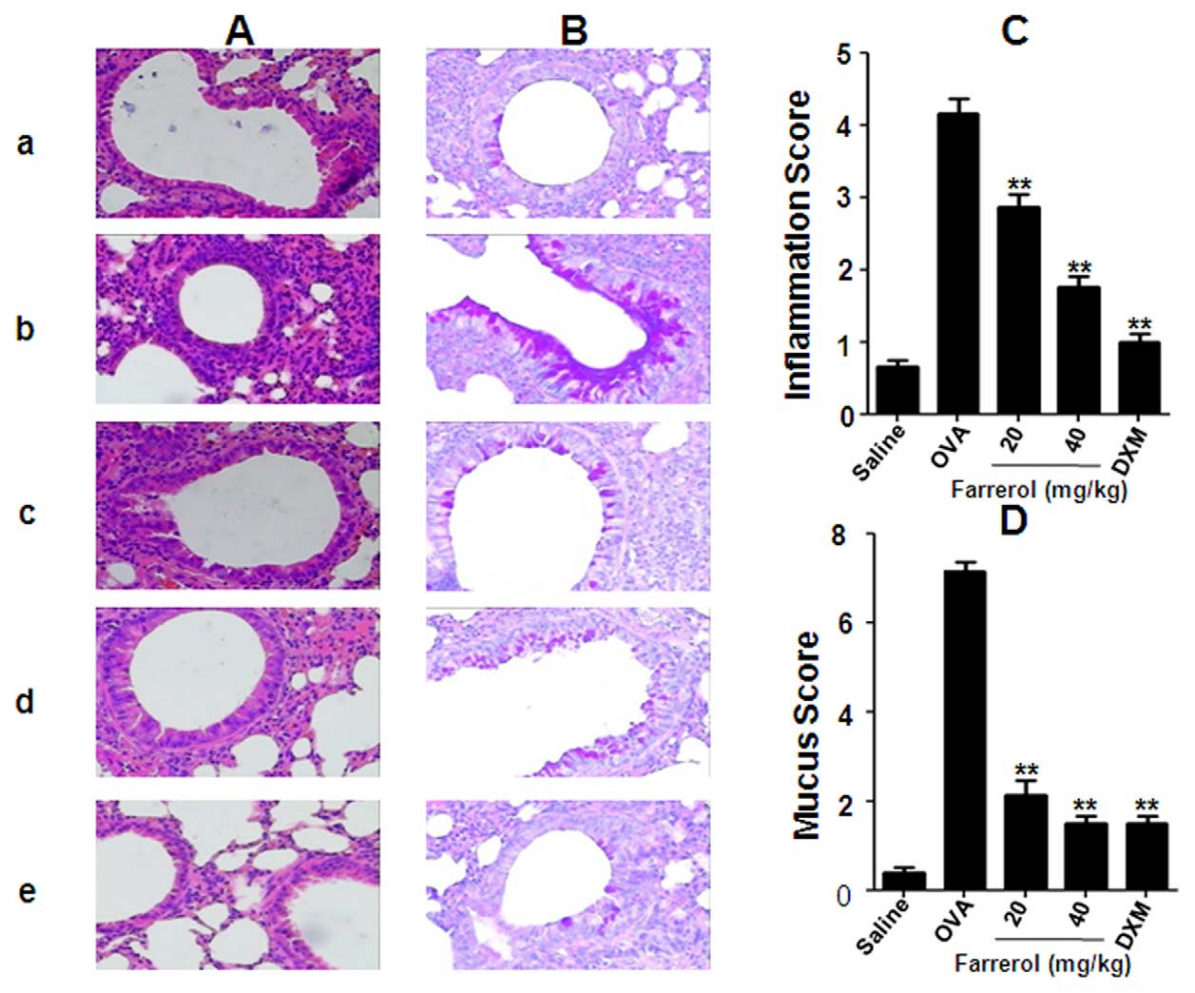
Effects of farrerol on lung tissue eosinophilia and mucus production. Histologic examination of lung tissue eosinophilia using HE staining (A) and mucus secretion using AB-PAS staining (B) from: (a) PBS-challenged mice; (b) OVA-challenged mice; (c) OVA-challenged mice treated with farrerol (20 mg/kg); (d) OVA-challenged mice treated with farrerol (40 mg/kg); (e) OVA-challenged mice treated with dexamethasone (2 mg/kg, magnification×400). Quantitative analyses of inflammatory cell infiltration (C) and mucus production (D) in lung sections were performed as previously described [35]. At least 5 different fields for each lung section was performed to score the inflammatory cells and goblet cells. Mean scores were obtained from 5 mice. **P < 0.01. vs. OVA.

To assess the anti-inflammatory effect of farrerol on the airway, histopathological studies were performed. Inflammatory cell infiltration in the peribronchial and perivascular areas (H&E staining), mucus overproduction and goblet cell hyperplasia (as detected by AB-PAS staining) were observed in OVA-challenged mice (**Fig. 3A and 3B**). Farrerol treatment (20 or 40 mg/kg) dose-dependently and markedly reduced the degree of inflammatory cell infiltration in the peribronchial and perivascular areas (**Fig. 3A and 3C**), mucus overproduction and goblet cell hyperplasia (**Fig. 3B and 3D**).

### Farrerol Regulates Ovalbumin-induced Bronchoalvelar Lavage Fluid T Helper 1 and T Helper 2 Cytokine Levels and Serum Immunoglobulin Production *in vivo*

**Figure 4:**
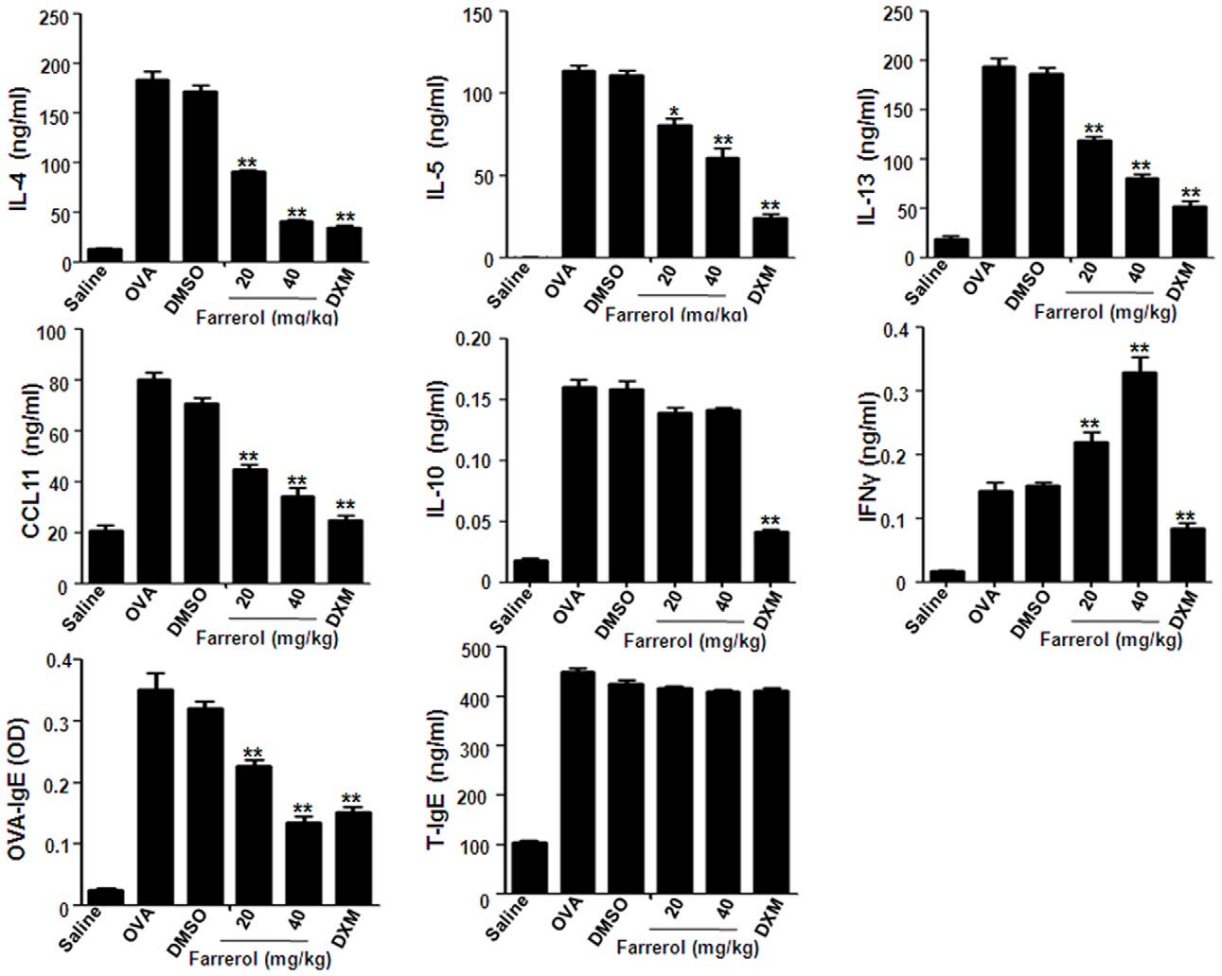
Effects of farrerol on cytokine and chemokine levels in BALF and serum immunoglobulin production in vivo. BALF and blood were collected and centrifuged 24 hours after the last OVA challenge, and the supernatants and serum were measured by ELISA. Results of IgE in serum (mean±SEM n = 10) are expressed as Optical Density values and are representative of at least three independent in vivo experiments. *p<0.05, **p<0.01 vs. OVA.

OVA-sensitized mice showed a notable increase in IFNγ, IL-10, IL-4, IL-5, IL-13 and eotaxin levels in the BAL fluid as compared with a saline aerosol control (**Figure 4**). Farrerol significantly reduced IL-4, IL-5, IL-13, CCL11 and increased IFNγ levels in a dose-dependent manner as compared with the DMSO control. However, there was no significant difference in IL-10 level which suggest farrerol can not suppress Th2 cytokines via upregulating IL-10. This finding suggested that farrerol can regulate the predominant Th1 and Th2 response in our OVA-induced mouse asthma model.

To further evaluate whether farrerol could modify an ongoing OVA-specific Th2 response in vivo, serum levels of total IgE and OVA-specific IgE were determined using ELISA. Farrerol (20 and 40 mg/kg) significantly suppressed OVA-specific IgE, which was induced by OVA.

### Effect of Farrerol on Ovalbumin-induced Airway Hyperresponsiveness

**Figure 5:**
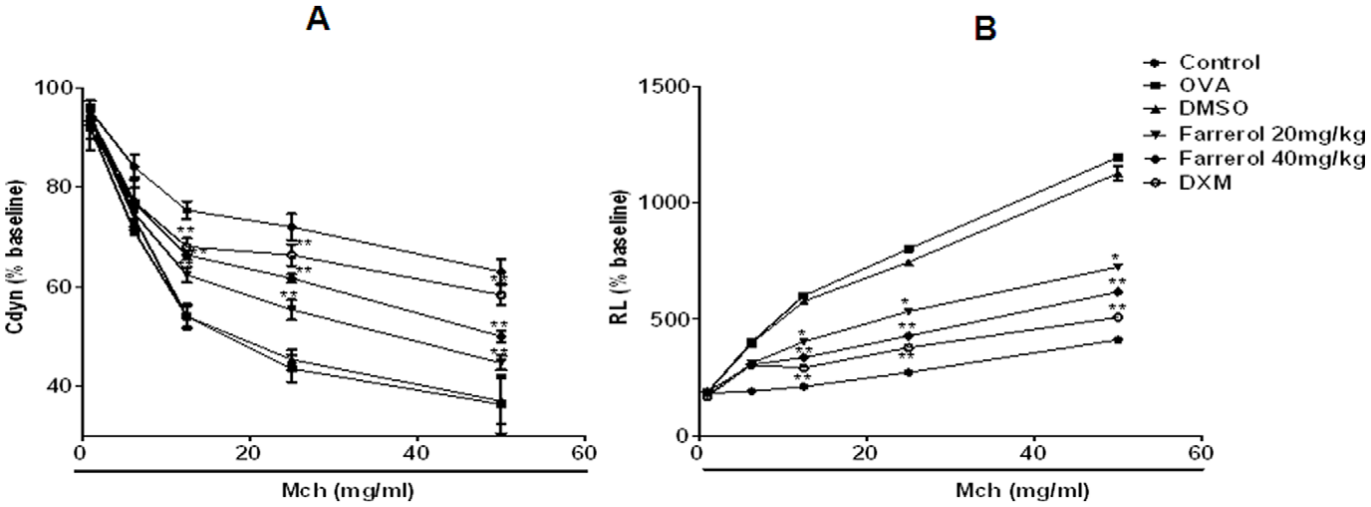
Effects of farrerol on OVA-induced AHR. Airway responsiveness of mechanically ventilated mice in response to aerosolized methacholine was measured 24 h after the last saline aerosol or OVA aerosol with pretreatment of either DMSO or Farrerol (20 and 40 mg/kg). AHR is expressed as percentage change from the baseline level of (A) dynamic compliance (Cdyn, n = 6 mice) and (B) lung resistance (RL, n = 5 mice). Cdyn refers to the distensibility of the lung and is defined as the change in volume of the lung produced by a change in pressure across the lung. RL is defined as the pressure driving respiration divided by flow. *p<0.05, **p<0.01 vs. OVA.

To investigate the effect of farrerol on AHR in response to increasing concentrations of methacholine, we measured both RL and Cdyn in mechanically ventilated mice. OVA-challenged mice developed AHR, typically reflected by a high RL and low Cdyn (**Figure 5**). Farrerol treatment (20 and 40 mg/kg) dramatically reduced RL and restored Cdyn in OVA-challenged mice in response to methacholine.

### Effect of Farrerol on OVA-induced Chemokines and Inflammatory Gene Expression in Allergic Airway Inflammation

**Figure 6:**
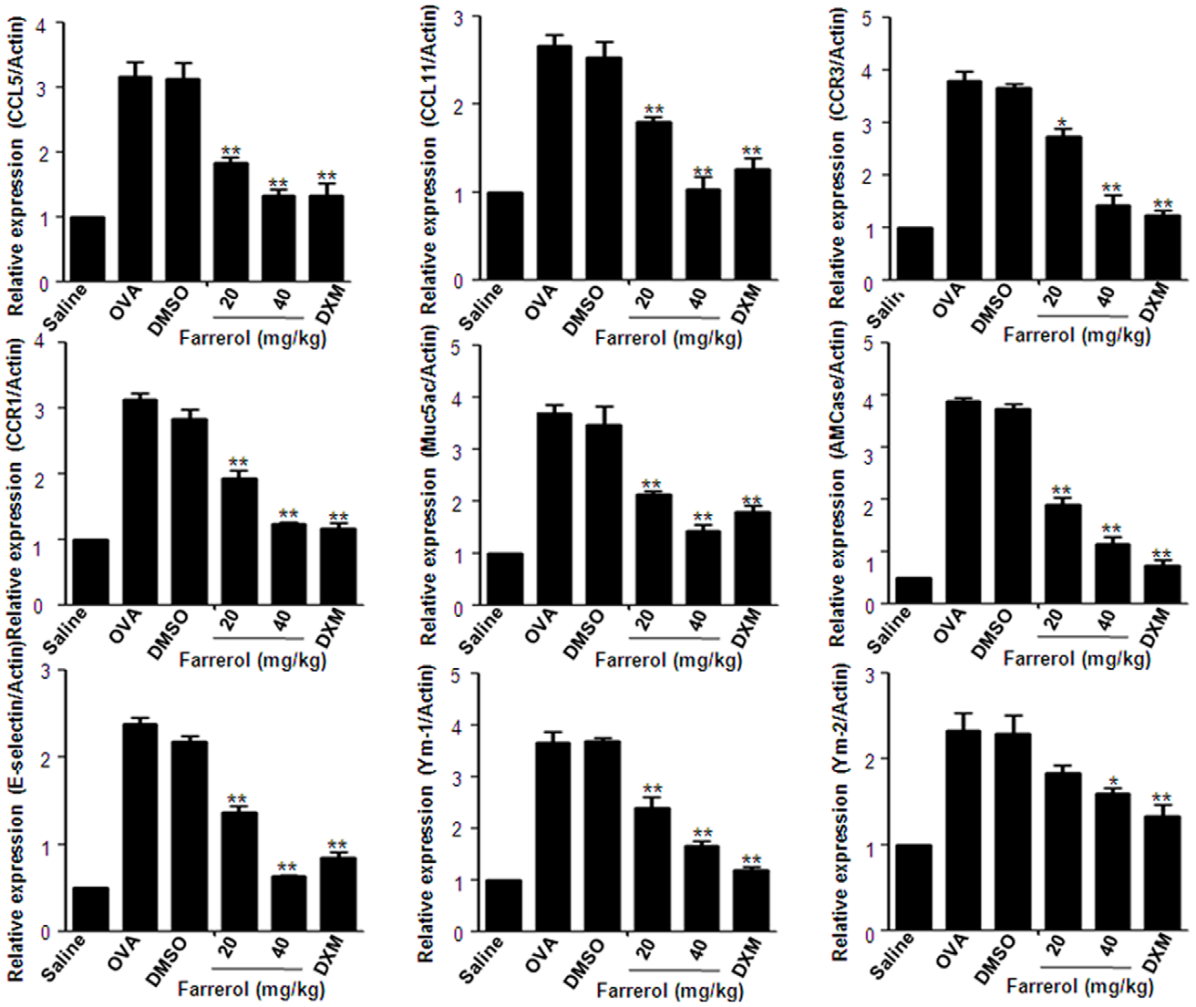
Effect of farrerol on OVA-induced inflammatory gene expression in allergic airway inflammation. Lung tissues were collected 24 hours after the last OVA aerosol challenge. The mRNA in whole-lung extracts was measured by real-time PCR. Treatment with farrerol (20 and 40 mg/kg) reduced OVA-induced mRNA expression in the lung. The values represent the mean±SEM of 10 animals in each group. **P<0.01. vs. OVA.

Whole-lung mRNA expression was determined using quantitative RNA analysis. The levels of the chemokines CCL5 and CCL11 and their receptors CCR1 and CCR3 were greatly enhanced in the OVA group in comparison with the control group. We found that farrerol treatment (20 and 40 mg/kg) significantly reduced the chemokines and the upregulation of their receptors after OVA challenge (**Fig. 6**).

Based on the above result that farrerol can inhibit the recruitment of inflammatory cells, airway inflammation and mucus hypersecretion, we further investigated the mRNA expression of E-selectin, AMCase, Ym1, Ym2 and Muc5ac, which have been shown to play vital roles in airway inflammation and remodeling. Pretreatment with farrerol also strongly suppress the expression of E-selectin, AMCase, Ym1, Ym2 and Muc5ac in the allergic airway.

### Effect of Farrerol on MAPK, Phosphatidylinositol 3-kinase Activation in vivo

**Figure 7:**
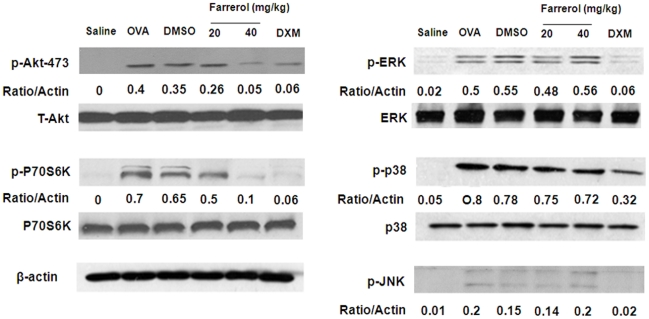
Effect of farrerol on Akt, P70S6K, and MAPK activation in vivo. Immunoblotting of Akt, P70S6K, and MAPK in proteins extracts of lung tissues isolated from mice 24 hours after the last OVA challenge pretreated with 20 or 40 mg/kg farrerol. β-actin was used as an internal control. Experiments were repeated three times and similar results were obtained.

To evaluate the effect of farrerol on the activation of MAPK and PI3K in the mouse asthma model, we homogenized the lung tissue to obtain a total protein lysate. The OVA-challenged mice showed significant upregulation of p-Akt, p-p70S6K, p-p38, pERK, and p-JNK. Farrerol can suppress the OVA-induced upregulation of p-Akt and p-p70 S6K in a dose-dependent manner. However, there was no significant downregulation of MAPK (**Fig. 7**).

### Effect of Farrerol on NF-κB Activation, IκBα Phosphorylation and Degradation in vivo

**Figure 8:**
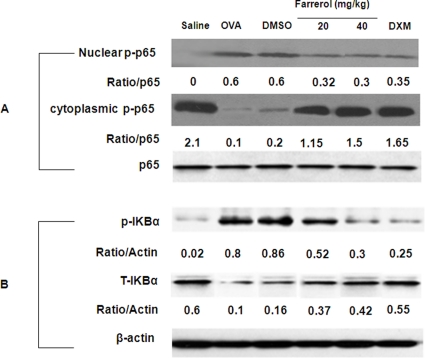
Effect of farrerol on the activation of NF-κB and IκBα phosphorylation and degradation in vivo. (A) Effect of farrerol treatment on nuclear translocation of NF-κB. Nuclear and cytoplasmic proteins from lung were analyzed by western blot with specific antibodies. (B) Effect of oxytetracycline treatment on IκBα phosphorylation and degradation. Total cellular proteins from lung were analyzed by western blot with specific antibodies. β-Actin was used as an internal control. Experiments were repeated three times and similar results were obtained.

To evaluate the effect of farrerol on NF-κB activation in a mouse asthma model, we homogenized the lung tissue to obtain total, nuclear and cytoplasmic proteins. In our study, **Fig. 8A** showed that OVA-challenged mice showed a notably increased nuclear protein level and a decreased cytoplasmic protein level, compared to PBS-challenged mice. Farrerol treatment significantly attenuated these alterations in a dose-dependent manner (*p<0.05, **p<0.01). To gain further insight into the mechanism of farrerol-mediated regulation of NF-κB in vivo, we examined the effect of farrerol on IκBα phosphorylation and degradation. As shown in **Fig. 8B**, OVA-induced IκBα degradation was significantly blocked by pretreatment with farrerol, which was related to IκBα phosphorylation.

### Effect of Farrerol on the Cytokines, PI3K and NF-κB Activation in vitro

**Figure 9:**
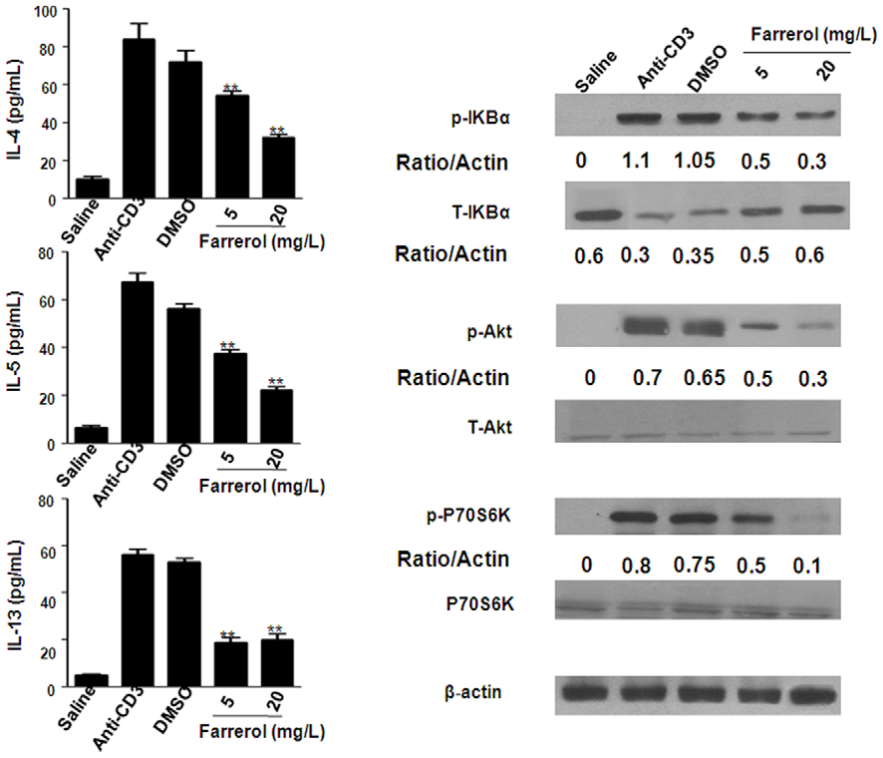
Effect of farrerol (20 and 40 mg/kg) on the IL-4, IL-5, and IL-13, Akt, P70S6K, and IκBα phosphorylation and degradation in vitro. The supernatant of Th2 cells was measured by sandwich ELISA. The values represent the mean±SEM of three independent in vitro experiments. Th2 cells were cultured with anti-CD3 (5 µg/ml) for 1 h (1 mg/L), total cellular proteins were analyzed by western blot with specific antibodies. β-Actin was used as an internal control. Experiments were repeated three times and similar results were obtained.

To assess whether farrerol treatment could directly affect lymphocyte function, we examined anti-CD3/CD28-challenged immune responses in Th2 cells. IL-4, IL-5, and IL-13 concentrations in the culture supernatant of Th2 cells were measured by sandwich ELISA (**Fig. 9A**). Treatment with farrerol (5 and 20 mg/L) can markedly decreased IL-4, IL-5 and IL-13 levels in the cell supernatant which induced by anti-CD3/CD28 (*p<0.05, **p<0.01). we also evaluated the effect of farrerol on p-Akt and the p-IκBα as a marker of NF-κB activation. We found that anti-CD3 stimulation can induce the expression of p-Akt, p-p70S6K and p- IκBα. As shown in Fig. 9B, pretreatment with farrerol downregulated p-Akt, p-p70S6K and IκBα phosphorylation and then block the of IκBα degradation.

### Effect of Farrerol on LPS-induced Acute Lung Injury

The total cell counts, differential cell counts, TNF-α, IL-6 and IL-8 in the BALF were evaluated 24 h after LPS administration. As shown in Fig. S1, LPS can markedly increase the number of total cells, neutrophils, macrophages, as well as the levels of TNF-α, IL-6 and IL-8 compared with the control group. However, farrerol does not have any effect on this increase compared to the dexamethasone group. Besides, farrerols can not decrease the upregulation of NF-κB, p-AKT, and MAPKs (**Fig. S2**).

## Discussion

Pulmonary disorders can be grouped according to whether the primary immune responses are characterized by innate or adaptive immune responses. In recent decades, using molecular, cellular assays together with knockout or transgenic animals, we have got more information about the immunological factors that contribute to the development pulmonary disorders. The immunologic features of pulmonary disorders can be used to categorize various conditions and provide focus for potential innovative therapies. Phytochemical and pharmacological studies have identified many potential anti-inflammatory substances, especially those derived from plants used in folk medicine, so natural products are becoming increasingly important as sources of pharmacotherapeutics, either for the treatment of infectious lung diseases or the theatment of noninfectious lung diseases [4], [20], [21]. In this study, we used two lung disease models: acute lung injury, characterized by innate immune responses and allergic asthma, involving adaptive immune responses, to demonstrate that farrerol can markedly reduce the allergic airway inflammation and AHR for allergic asthma model, but has no effect in an acute lung injury model.

Airway inflammation is a natural response of the lung to the presence of internal and external substances, which is characterized by innate immunity and adaptive immunity. Asthma is characterized by AHR and chronic airway inflammation, which includes the infiltration of inflammatory cells into lung tissues, mucus overproduction, allergen-specific IgE, the over-expression of Th2-mediated cytokines, including interleukin (IL)-4, IL-5 and IL-13, and chemokines such as CCL11 (CCL11) and RANTES (CCL5). In contrast, Th1 cytokines such as interferon-γ (IFN-γ), can inhibit the development of allergic lung inflammation through down-regulating Th2 responses [22]. The predominant inflammatory cell recruited into asthmatic lung tissue is the eosinophil, which is associated with the production of IL-5. However, macrophages, the minor inflammatory cell recruited into asthmatic lung tissue, can produce IL-10 to suppress Th2 cytokine production and eosinophilia but augments airway reactivity. Acute lung injury is characterized by systemic airway inflammatory response including cytokines (e.g., TNF-α, IL-6, IL-8), chemokines, pro-inflammatory mediators and a variety of cells, which regulate the migration and pulmonary infiltration of neutrophils into the interstitial tissue [23]. Neutrophils are an important component of the inflammatory response that characterizes acute lung injury (ALI) [24]. In addition, PI3K contribute to the pathogenesis of asthma or acute lung injury by influencing the recruitment of eosinophils or neutrophils [12]. In previous report, farrerol has been reported to block FBS-induced vascular smooth muscle cells proliferation by acting as a estrogen receptors agonist [25]. In addition, estrogen has been shown to aggravates inflammation in pseudomonas aeruginosa pneumonia in cystic fibrosis mice via increasing the total white blood cell counts and neutrophils, Th1 cytokines (TNF-α and IL-6) Th2 cytokines (IL-5) and extaxin [26]. From our study, we can conclude that farrerol can reduce the number of total inflammatory cells and eosinophils in BAL fluid; IL-4, IL-5 and IL-13 levels in BAL fluid, OVA-specific IgE levels in serum; mRNA levels of CCL5, CCL11, CCR3, CCR5, E-selectin, chitinases, and Muc5ac in lung tissues. Furthermore, as IFN-γ was evoked by farrerol in the lung of mice this cytokine may act to further suppress type 2 pulmonary inflammation. After OVA challenge, farrerol can also inhibit the expression of p-Akt. However, farrerol does not have any effect on TNF-α, IL-6, and IL-8, which are induced in acute lung injury. So these result suggested that on the one hand, farrerol maybe act as an estrogen receptors agonist to result in aggravating the inflammation in infectious inflammation model; on the other hand, farrerol play an important role in reducing airway inflammation in uninfectious inflammation model. The distinct effect of farrerol on the different airway inflammation maybe depending on its potential as an estrogen receptors or not.

The transcription factor NF-κB regulates the expression of a large number of genes in response to infection, inflammation and other endogenous and exogenous stressors. Under resting conditions, NF-κB is held inactiveby IκB. However, NF-κB can be activated by some stimulation of various receptors including TNF receptor, Toll-like receptors (TLRs) and T-cell receptor (TCR). Persistent activation of NF-κB is central to the pathogenesis of many inflammatory lung disorders including chronic obstructive pulmonary, asthma, pneumonia, and acute lung injury. In our study, farrerol can inhibit NF-κB activation induced by OVA in an allergic asthma model, but not in acute lung injury model, which suggests that the decreased NF-κB activation could account for the inhibition of airway inflammation and AHR.

Corticosteriods have a puissant anti-inflammatory action and have a central role in the treatment for asthma [27]. However, prolonged use of corticosteriods, especially at higher doses, has been accompanied by concerns about both systemic and local side effects [28]. Date in this study indicates a potential therapeutic value for farrerol in the treatment of allergic asthma. In the context of current treatments for allergic asthma, future experimental studies are needed to address if farrerol can reduce corticosteriods dosage using combinations of both drugs.

## Materials and Methods

### Reagents

The mAbs against mouse CD3, CD28, IL-4, IL-5, IL-13, TNFα, IL-6, IL-8, IFN-γ and IL-10 ELISA kits were purchased from Biolegend (California, USA). CCL11 mouse ELISA kit was purchased from Abcam (CA, USA). Western blot antibodies were purchased from Santa Cruz (Santa Cruz, CA, USA) and Cell Signaling Technology Inc. (Beverly, MA). Dimethy sulfoxide (DMSO) and OVA (Grade V) were purchased from Sigma Chemical Co. (St. Louis, MO, USA). Farrerol (analytical grade, purity ≥98%) was obtained from the National Institute for the Control of Pharmaceutical and Biological Products (Beijing, China).

### Animals

Female BALB/c mice, weighing approximately 18 to 20 g, were purchased from Shanghai Jingke Industrial Co., LTD (Certificate SCXK2003-0003) (Shanghai, China) and bred under specific pathogen-free conditions. The mice were housed in microisolator cages and received food and water ad libitum. All animal studies were conducted according to the experimental practices and standards approved by the Animal Welfare and Research Ethics Committee at Jilin University.

### Sensitization and Challenge with OVA and Treatment

These mice were divided into six groups (n = 10) and were sensitized with 20 µg ovalbumin adsorbed in 100 µg/ml of Imject Alum by i.p. injection on days 0, 7, 14 (general sensitization) in all mice. On day 14, mice are anesthetized and 100 µg of OVA in 50 µl of PBS administered intranasally in all mice except negative control sensitized with PBS. Mice are again anesthetized before being challenged with 50 µg of OVA in 50 µl of PBS on each of days 25–27. On days 25–27, farrerol at 20 and 40 mg/kg and dexamethasone (2 mg/kg) were given by i.p. injection 1 h prior to OVA administration.

### Murine Model of LPS-induced ALI

LPS-induced ALI was induced as previously described [29]. Briefly, mice were anesthetized and challenged with intranasal 10 µg of LPS in 50 µl PBS. Control mice were given 50 µl PBS i.n. instillation without LPS. Farrerol at 20 and 40 mg/kg and dexamethasone was i.p. injected 1 h prior to LPS administration.

### Collection of Blood and Bronchoalveolar Lavage

At selected times after the last inhalational exposure, mice were anesthetized and bled via the brachial plexus for the collection of blood samples used to estimate the levels of total IgE and OVA-specific IgE. Bronchoalveolar lavage (BAL) was performed twice by intratracheal instillation of 500 µl of PBS. The lavage fluid was centrifuged and the supernatants were used for cytokine and chemokines measurements. Cell pellets were resuspended in 1 ml of PBS and used for total and differential cell counts as described [30].

### Lung Histology

Histopathological evaluations were performed on mice. Left lungs were removed by dissection and fixed in 4% paraformaldehyde. Lung tissues were sectioned, embedded in paraffin, and cut into 3 µm sections. Tissue sections were then stained with hematoxylin and eosin stain (H&E) for general morphology [31] and with AB-PAS for the identification of goblet cells in the epithelium [32].

### Measurements of Airway Hyperresponsiveness

Airway responsiveness was assessed by methacholine-induced airway resistance (RL) and lung compliance (Cdyn) using a whole-body plethysmograph chamber (Buxco, Sharon, CT) as described [33]. Mice were anesthetized with pentobarbital sodium (100 mg/kg) and tracheotomy was performed as described [33]. The internal jugular vein was cannulated and connected to a microsyringe for aerosolized methacholine administration. Baseline lung resistance, dynamic compliance, and responses to aerosolized saline (0.9% Nacl) were measured first, followed by responses to increasing dose (6.25 to 50 mg/ml) of aerosolized methacholine. Results are expressed as a percentage of the respective basal values.

### RNA Preparation and Quantitative RT-PCR

Total RNA was isolated from lungs (5 mice in each group) using the TRIzol reagent (SIGMA-Aldrich, St. Louis, USA). Real-time PCR was performed on cDNA samples using the SYBR Green system (Bio-Rad; Richmond, CA). Primers for inflammatory bio-markers are shown in Table 1. Analysis was performed using the sequence detection software supplied with the instrument. A melting curve analysis was performed to control for the specificity of the amplification products.

**Table 1:** Primer sets for reverse transcriptase-polymerase chain reacion analysis.

Targets	Forward	Reverse
CCL11	AAACCATAAACAACCTCCTC	CAATAATCCCACATCTCCTT
CCL5	GGATAGAGGGTTTCTTGATT	GCTGATTTCTTGGGTTTG
CCR1	CACTCACCGTACCTGTAGCC	TCTGATGATCCCTGCATAGC
CCR3	TCTGCTGAGATGTCCCAATA	TCACCAACAAAGGCGTAG
MUC-5ac	GTGTCGGCCGGAGAAAGTTGGT	GTCCTGTTGAGCCTGGCCTGTG
Ym-1	CCAGCAGAAGCTCTCCAGAAGCA	CAGCTGGTAGGAAGATCCCAGCTGT
Ym-2	TCCACTTTGAACCACATTCCAAGGC	CGAGAGACTGAGACAGTTCAGGGA
AMCase-1	TGGACACACCTTCATCCTGA	CCTCAGTGGCTCCACTTCTC
E-selecin	CCCTTCCACAGAACCTACCA	TCAGCAGACATTGCTTCACC
β-Actin	CTGTCCCTGTATGCCTCTG	ATGTCACGCACGATTTCC

### CD4+ T Cell Isolation and Th2 Differentiation

To obtain naive CD4+T cells, single cell suspensions were prepared from spleens and treated with monoclonal antibodies to CD4 coupled to magnetic beads according to the protocol provided by the manufacturer. For Th2 differentiation, IL-2 (10 ng/ml), IL-4 (5 ng/ml) and anti-IFN-γ (4 µg/ml) were added.

### Cytokine Assays

Naive CD4+ T cells were stimulated for 72 h with anti-CD3 (5 µg/ml) plus anti-CD28 (2.5 µg/ml) and Th2 differentiated cells were restimulated for 24 h with anti-CD3 (5 µg/ml) in the presence/absence of farrerol (5 and 20 mg/l), after which supernatants were collected and cytokine concentrations were measured by ELISA using commercially available kits. Control cells were incubated in medium alone for the duration of the experiment. ELISA was also used to determine the levels of Th2 cytokines in BALF.

### Western Blot Analysis of MAPK, NF-κB and PI3K

Lung tissues were added to lysis buffer and homogenized. Th2 cells (1×10^6^) were plated into 6-well plates and pretreated with 5 and 20 mg/L of farrerol, 1 h prior to a 1 h treatment with anti-CD3 (5 µg/ml). Nuclear and cytoplasmic fractions of lung were prepared as previously described [34]. Whole cell lysates were prepared in 1% Non-diet P-40 lysis buffer with freshly added protease and phosphatase inhibitors. Protein were separated by SDS/PAGE and transferred onto PVDF membrane. Membranes were probed for different antibodies.

### Statistical Analysis

All values were expressed as means±the standard error of the mean (SEM). Differences between mean values of normally distributed data were assessed by the two-tailed Student t test. Statistical difference was accepted at P<0.05.

## Supporting Information

Figure S1 **Effect of farrerol on production of inflammatory cytokines TNF-α, IL-6 and IL-8 in the BALF of LPS-induced ALI mice.** Mice were given an oral administration of farrerol 1 h prior to an i.n. administration of LPS. BALF was collected at 6, 12 and 24 h following LPS challenge to analyze the inflammatory cytokines TNF-α, IL-6 and IL-8. The values presented are the means±SEM (n = 5 in each group). *p<0.05 vs. LPS group;
(TIF)

Figure S2 **Effect of farrerol on Akt, P70S6K, NF-κB and MAPK activation in vivo.** Immunoblotting of Akt, NF-κB, P70S6K, and MAPK in proteins extracts of lung tissues isolated from mice 24 hours after the LPS challenge pretreated with 20 or 40 mg/kg farrerol. β-actin was used as an internal control. Experiments were repeated three times and similar results were obtained.
(TIF)
