# Supplementary figures and images for: Different Effects of Farrerol on an OVA-Induced Allergic Asthma and LPS-induced Acute Lung Injury
Source: PLoS One. 2012 Apr 26;7(4):e34634. doi: 10.1371/journal.pone.0034634 (PMC3338508; doi:10.1371/journal.pone.0034634)

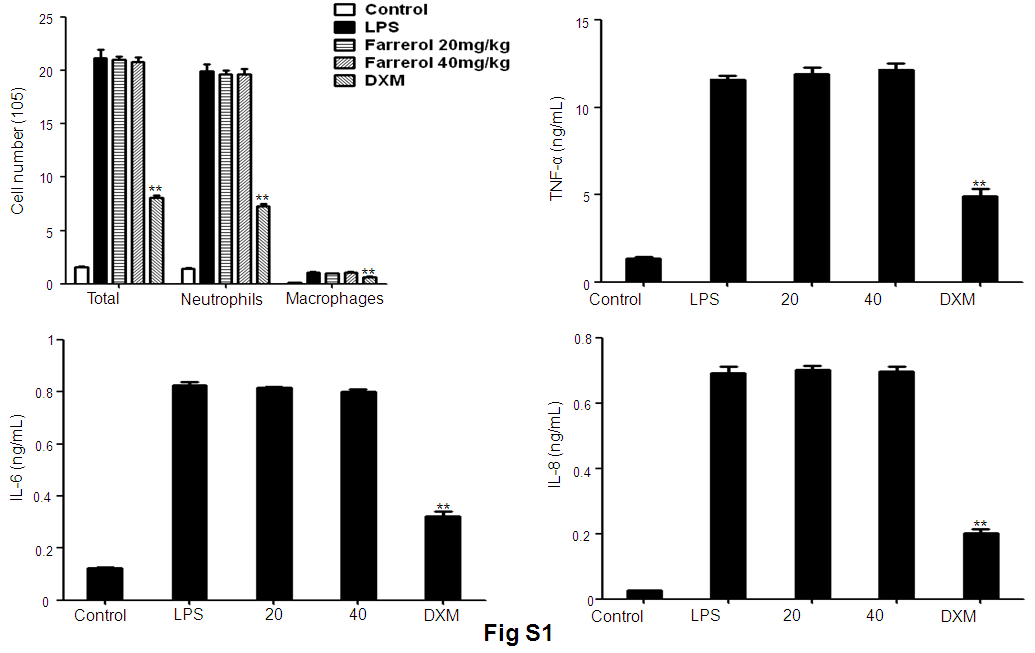

Supplement: Figure S1 — Effect of farrerol on production of inflammatory cytokines TNF-α, IL-6 and IL-8 in the BALF of LPS-induced ALI mice. Mice were given an oral administration of farrerol 1 h prior to an i.n. administration of LPS. BALF was collected at 6, 12 and 24 h following LPS challenge to analyze the inflammatory cytokines TNF-α, IL-6 and IL-8. The values presented are the means±SEM (n = 5 in each group). *p<0.05 vs. LPS group; (TIF) [file pone.0034634.s001.tif]

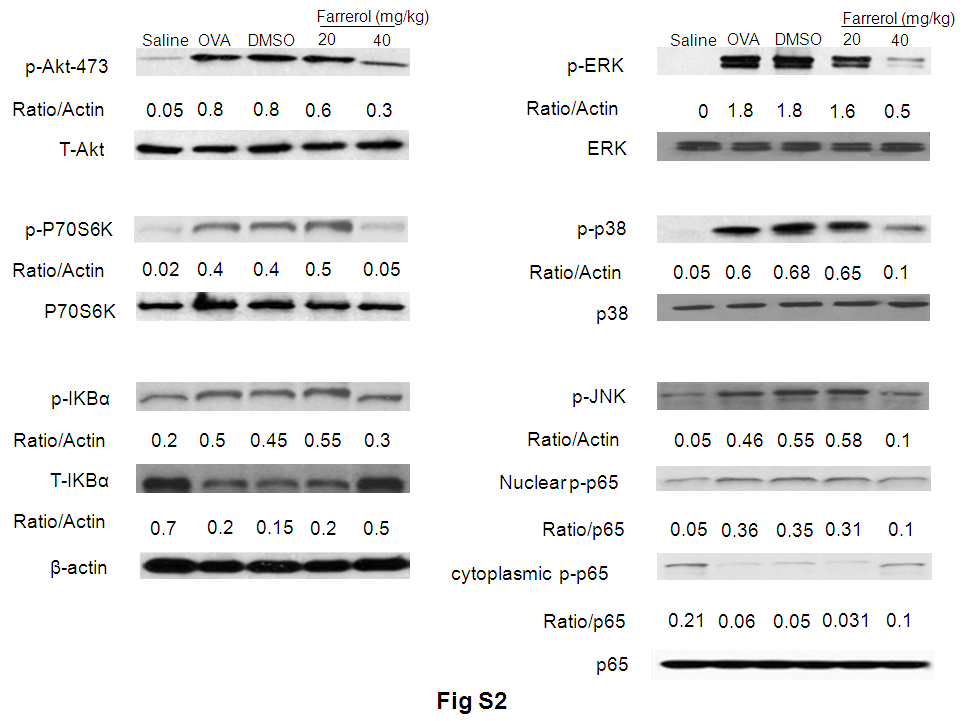

Supplement: Figure S2 — Effect of farrerol on Akt, P70S6K, NF-κB and MAPK activation in vivo. Immunoblotting of Akt, NF-κB, P70S6K, and MAPK in proteins extracts of lung tissues isolated from mice 24 hours after the LPS challenge pretreated with 20 or 40 mg/kg farrerol. β-actin was used as an internal control. Experiments were repeated three times and similar results were obtained. (TIF) [file pone.0034634.s002.tif]
